# MIDAS2: Metagenomic Intra-species Diversity Analysis System

**DOI:** 10.1093/bioinformatics/btac713

**Published:** 2022-11-02

**Authors:** Chunyu Zhao, Boris Dimitrov, Miriam Goldman, Stephen Nayfach, Katherine S Pollard

**Affiliations:** Data Science, Chan Zuckerberg Biohub, San Francisco, CA 94158, USA; Gladstone Institute of Data Science and Biotechnology, Gladstone Institutes, San Francisco, CA 94158, USA; Chan Zuckerberg Initiative, Redwood City, CA 94063, USA; Gladstone Institute of Data Science and Biotechnology, Gladstone Institutes, San Francisco, CA 94158, USA; Biomedical Informatics Graduate Program, University of California San Francisco, San Francisco, CA 94158, USA; Department of Energy, Joint Genome Institute, Berkeley, CA 94720, USA; Environmental Genomics and Systems Biology Division, Lawrence Berkeley National Laboratory, Berkeley, CA 94720, USA; Data Science, Chan Zuckerberg Biohub, San Francisco, CA 94158, USA; Gladstone Institute of Data Science and Biotechnology, Gladstone Institutes, San Francisco, CA 94158, USA; Department of Epidemiology and Biostatistics, University of California, San Francisco, CA 94158, USA

## Abstract

**Summary:**

The Metagenomic Intra-Species Diversity Analysis System (MIDAS) is a scalable metagenomic pipeline that identifies single nucleotide variants (SNVs) and gene copy number variants in microbial populations. Here, we present MIDAS2, which addresses the computational challenges presented by increasingly large reference genome databases, while adding functionality for building custom databases and leveraging paired-end reads to improve SNV accuracy. This fast and scalable reengineering of the MIDAS pipeline enables thousands of metagenomic samples to be efficiently genotyped.

**Availability and implementation:**

The source code is available at https://github.com/czbiohub/MIDAS2.

**Supplementary information:**

Supplementary data are available at *Bioinformatics* online.

## 1 Introduction

Metagenotyping, the identification of intraspecific genetic variants in metagenomic data, is a powerful approach to characterizing population genetic diversity in microbiomes. Most pipelines identify variants based on alignment of reads to reference databases of microbial genomes and/or gene sequences (Supplementary Fig. S1). While comprehensive reference databases can reveal strain-level relationships which would be otherwise overlooked (Beghini *et al.*, 2021), alignment to large databases is computationally intensive. Furthermore, the divergence of reference genomes from strains in the metagenomic sample affects sensitivity and precision (Bush *et al.*, 2020; Olm *et al.*, 2021), and existing metagenotyping tools do not automatically adapt database files based on information in the metagenome. In this article, we introduce Metagenomic Intra-Species Diversity Analysis System (MIDAS2) (Supplementary Fig. S2), a major update to MIDAS (Nayfach *et al.*, 2016) (Supplementary Table S1) that addresses these challenges through (i) a new database infrastructure geared to run on AWS Batch and S3 that achieves elastic scaling for constructing database files from large collections of genomes; and (ii) a fast and scalable implementation of the single nucleotide variant (SNV) calling pipeline that enables metagenotyping in thousands of samples with improved accuracy achieved through utilization of paired-end reads and databases customized to the species present in the samples. As the only tool that integrates all steps of the metagenotyping process, from database customization to alignment and variant calling, MIDAS2 helps to promote reproducible research.

## 2 Implementation

We generated MIDAS Reference Databases (MIDAS DB), comprised of species pangenomes, marker genes and representative genomes, from two public microbial genome collections: UHGG v.1 (Almeida *et al.*, 2021) (4644 species/286 997 genomes) and GTDB v202 (Parks *et al.*, 2022) (47 893 species/258 405 genomes). This is a significant increase in database content compared to MIDAS DB v1.2 (5952 species/31 007 genomes) and other tools (Supplementary Table S2). We implemented a new infrastructure that dramatically simplifies building a new MIDAS DB for other genome collections by using a table-of-contents file assigning genomes to species and denoting the representative genome for each species (Supplementary Fig. S3). MIDAS DBs can be built locally, which enables customized selection of representative genomes, a key component of accurate SNV calling.

Metagenotyping SNVs across large numbers of samples is computationally intensive. First, alignment and pileup are applied to each species in each sample (single-sample step) without assuming a single strain per sample. Then these pileup results must be scanned for each genomic site to compute population SNVs (across-samples step). Previously published methods cap the number of processors (CPUs) that can be used, because they parallelize over the number of species being genotyped (Supplementary Note). The SNV module of MIDAS2 achieves better CPU utilization by splitting genomic sites into multiple chunks per species. We execute parallelization over chunks in a way that does not destroy cache coherence to the point where computation stalls on input or output (I/O; Supplementary Note).

## 3 Results

**Fig. 1.**
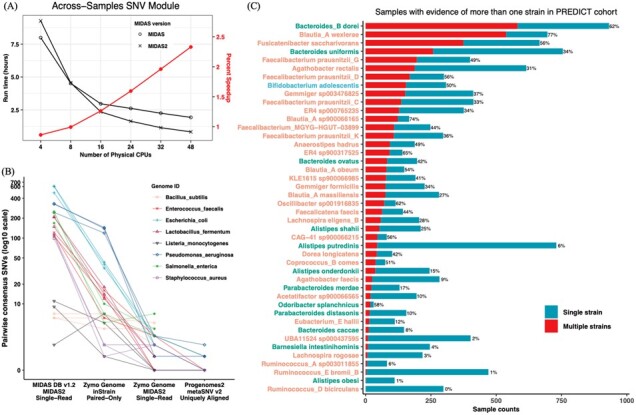
Speed, accuracy and application of MIDAS2. (**A**) The SNV module of MIDAS2 was re-engineered to parallelize within species, making it increasingly faster than MIDAS as we deploy more CPUs. This analysis was performed with 211 metagenomic samples (NCBI accession: PRJNA400072). (**B**) Metagenotype accuracy was benchmarked using identical aliquots of a standardized microbial community, for which all consensus SNVs are false positives. More errors are made with a large reference genome database compared to one with only the species in the community (MIDASDB v1.2 versus Zymo Genome). Post-alignment filters, including how paired-end reads are handled, differ between tools (run with default filters) and affect false positive rates. Despite a large database (Pangenomes2), metaSNV v2 has a low false positive rate due to using only uniquely aligned reads, but this comes with the cost of lower sensitivity. Supplementary Figure S6 shows how database and post-alignment filters affect errors in population SNVs; MIDAS2 and inStrain have similar error rates with Zymo Genomes. (**C**) Distribution of samples with evidence of a strain mixture versus one dominant strain for 44 species metagenotyped by MIDAS2 in 1097 samples from the PREDICT cohort (NCBI accession: PRJEB39223)

We compared the running time and memory utilization of the single-sample and across-samples SNV modules of MIDAS and MIDAS2, using the same database (MIDAS DB v1.2) and 211 samples from an inflammatory bowel disease cohort (NCBI accession: PRJNA400072). The single-sample SNV module of MIDAS2 is slightly faster than MIDAS (Supplementary Fig. S4), with database customization and Bowtie2 alignment taking up to 75% of run time (Supplementary Fig. S5). The across-samples SNV module benefited more from parallelization, scaling linearly (Supplementary Fig. S4) and running 2.33 times faster in MIDAS2 with 48 CPUs (Fig. 1A). We also compared runtime with inStrain v1.6.3 Olm *et al.* (2021) (Supplementary Table S13) and metaSNV v2 Van Rossum *et al.* (2022) (Supplementary Table S14).

MIDAS2, inStrain and metaSNV v2 were applied to three aliquots of a standardized bacterial community (Olm *et al.*, 2021), and SNVs were compared between aliquots which should have identical metagenotypes (Supplementary Note). metaSNV v2 has the fewest false positives by only using uniquely aligned reads, but it genotyped just five of the eight species in the community (Supplementary Table S5). InStrain and MIDAS2 correctly detected all eight species. When both are run with a genome database containing only the reference genomes of the strains in the community, MIDAS2 has fewer false positives (Fig. 1B). However, the false positive rate of MIDAS2 is higher when using the MIDAS DB v1.2, in which these species’ reference genomes are diverged from the sample. Thus, high-quality reference genomes and post-alignment filters that balance false positives against false negatives are crucial for metagenotyping.

Since metaSNV v2 was previously shown to be efficient enough to metagenotype thousands of samples, we assessed the scalability of MIDAS2 compared to metaSNV v2 on 1097 samples from the PREDICT study (NCBI accession: PRJEB39223), using MIDAS DB UHGG with both tools (Supplementary Note). Despite the same species selection criteria, MIDAS2 metagenotyped many more species (44 versus 14 for metaSNV v2) (Supplementary Note). MIDAS2 used more memory (21.21 GB versus 4 GB peak RAM utilization) and ran slightly longer (average 106 versus 84 min per species) to achieve this. We conclude that MIDAS2 can metagenotype thousands of samples with reasonable computational costs, providing a more sensitive alternative to metaSNV v2.

For each of the 44 species from PREDICT with MIDAS2 metagenotypes, we quantified evidence of a single dominant strain versus mixtures of multiple strains in each sample with an existing method (Garud *et al.*, 2019). While most species showed evidence of distinct lineages across samples (Supplementary Fig. S8), single samples often had a single dominant strain (Fig. 1C). However, samples with strain mixtures were common for several species, including *Bacteroides_B dorei* (62%) and *Faecalibacterium prausnitzii_G* (49%) (Supplementary Figs S9 and S10). We also showed that MIDAS2 can detect simulated strain mixtures with high accuracy (Supplementary Table S15), lending credibility to this finding.

## Funding

This work was funded by the Chan Zuckerberg Biohub, Gladstone Institutes, NHLBI [HL160862], and NSF [1563159].

*Conflict of Interest*: none declared.

## Supplementary Material

btac713_Supplementary_Data
