# Supplementary material for: MIDAS2: Metagenomic Intra-species Diversity Analysis System
Source: Bioinformatics. 2022 Nov 2;39(1):btac713. doi: 10.1093/bioinformatics/btac713 (PMC9805558; doi:10.1093/bioinformatics/btac713)
Supplement: btac713_Supplementary_Data [file btac713_supplementary_data.zip › btac713_Supplementary_Data/supplementary.pdf]

Supplementary Note

MIDAS2: Strain-level Reads-to-Table Metagenotyping Pipeline

MIDAS2 performs strain-level metagenomics analysis through a SNV module and a CNV module. Each module includes two sequential steps: single-sample analysis and across-samples integration of single-sample results to identify population variants (Supplementary Figure S2). The SNV module is based on alignment of metagenomic reads to one representative genome per species (rep-genomes), and the CNV module is based on alignment of reads to pan-genomes; both use an estimate of which species are present in the sample to customize the sample-specific reference database.

Given a collection of shotgun metagenomics samples, both the SNV and CNV module start with identifying the list of species that are sufficiently abundant for metagenotyping, based on profiling 15 universal, single copy genes (SCGs). By default, all species with median SCG coverage > 2X are metagenotyped. If the purpose is to genotype low abundance species, users are responsible for adjusting the cutoff (e.g., median SCGs coverage > 0). Species to be metagenotyped are then used to construct a sample-specific rep-genome database that only contains the representative genomes for these species (SNV module) and/or a pan-genome database that only contains the pan-genomes for these species (CNV module). This customization of a pre-computed MIDAS DB to the sample improves metagenotype accuracy by reducing "cross-mapping", whereby metagenomic sequencing reads align erroneously to conserved regions in the genomes of closely related species.

The SNV module starts with single-sample pileup, by aligning reads to a sample-specific rep-genome database. The alignment results for all the species in the rep-genome database are reported, such as *fraction_covered mean_coverage*, and *mapped_reads*. MIDAS2 purposely holds off on any filtering or species selection with the single-sample pileup results until across-samples SNV analyses are performed. Upon the completion of the single-sample SNV analyses for all the samples, MIDAS2 will compute the population SNVs across all the samples (see Population SNV Computation section).

It is worth noting that the MIDAS2 single-sample SNV module is not designed for consensus SNV calling, and by design MIDAS2 does not apply any species or variants calling filters at the single-sample level. The default output is the count of reads for each nucleotide (A, C, T, G) for all sites covered by more than two reads (i.e., MIDAS2 does not assume a single strain per sample). However, in recognition of the need for single-sample consensus SNV calling, we added an *--advanced* mode to the single-sample SNV analysis in MIDAS2 to report per species major allele and minor allele for all the genomic sites covered by at least two reads, upon which any variant calling filter would be applied by the users. We highly recommend users set the parameter *--ignore_ambiguous* to ignore ambiguous alleles, where genomic sites recruit tied read counts.

The CNV module starts with single-sample alignment of reads to the sample-specific pan-genome database. As in the SNV module, this only includes the pan-genomes of species being genotyped (i.e., sufficiently abundant species based on median SCGs coverage). The alignment summaries for all the species in the customized pan-genome database are reported (e.g., *mean_coverage, mapped_reads*), and these are used to determine the scope of the across-samples CNV analysis. For example, only species that pass vertical pan-gene read depth > 1X (by default) are included in the across-samples CNV computation. For each gene in each species, coverage is normalized by the mean coverage of all the SCGs to estimate copy number per cell (Nayfach and Pollard 2016). A brief comparison of the CNV module with other methods can be found in Supplementary Table S3.

Features Distinguishing Different Reference-based Metagenotyping Pipelines

All read mapping based metagenotyping pipelines share an underlying structure, yet they differ from one another in the availability of genome databases, the customization of reference genomes to the species that are present in the sample, read aligners, pileup tools, post-alignment filters, and the contents of input and output files (Supplementary Figure S1).

Database. Whole-genomes are used as the reference for most pipelines (MIDAS2, metaSNV v2 (Van Rossum et al. 2021), InStrain (Olm et al. 2021)), while species-specific marker genes are used by StrainPhlan (Truong et al. 2017). MIDAS2 is the only pipeline providing precomputed reference genome databases: namely MIDAS DB UHGG and MIDAS DB GTDB. MIDAS2 builds a database of representative genomes (rep-genomes) customized to only include species that are sufficiently abundant in the sample, which is determined via coverage of 15 SCGs. In contrast, metaSNV v2 and inStrain leave database selection and customization to their users.

Alignment and pileup. MIDAS2 and inStrain provide post-alignment filters based on the read alignment quality (e.g., alignment sequence similarity and uniqueness), while metaSNV v2 leaves this step to the user. MIDAS2 performs read pileup directly from counting reads covering each nucleotide (*count_coverage*), while metaSNV v2 and inStrain both use samtools mpileup (Danecek et al. 2021) or pysam (Andreas Heger et. al. 2021) for the pileup task. See Supplementary Table S12 for the resulting differences in variant calling results. One possible reason for differences in metagenotypes is that samtools does not use soft-clipped reads. This may be desirable for certains applications. Yet since all microbiome pipelines apply post-alignment filtering to the raw alignment file before the pileup and variant calls, an unfiltered read pileup seems to be appropriate, so this is what we implemented in MIDAS2.

Genotypes. For single-sample SNV calling, inStrain only reports non-reference variants (when the major allele is different from the reference allele), which can cause missing allele information for downstream across-samples population SNV calling. MIDAS2 reports single-sample pileup results for all genomic sites covered by at least 2 reads. Therefore, MIDAS2 can avoid repeating single-sample pileup when more samples are added to the analysis. For across sample results, MIDAS2 reports population SNVs of all genomic sites meeting the user-defined types (e.g., *snp_type*), while inStrain compares pairs of samples. On the other hand, metaSNV v2 does not report any single-sample pileup results, and only reports the across-samples non-reference variants. Adding samples to an existing analysis, metaSNV v2 therefore has to be run again from the beginning.

Parallelization. MIDAS and metaSNV v2 parallelize computations over species, as does the published version of inStrain (Olm et al. 2021). MIDAS2 parallelizes over segments of each species' genome (see Chunkified pileup implementation), the sizes of which are chosen to optimize CPUs utilization. A recent release of inStrain (v1.6.3) also offers parallelization within species.

In summary, MIDAS2 is the only reads-to-table metagenotyping pipeline. By integrating and automating all steps of the metagenotyping process, MIDAS2 helps to promote reproducible research.

Population SNV Computation

Population SNV analysis (across-samples step) restricts attention to "sufficiently well" covered species in "sufficiently many" samples. To be specific, a given <*species, sample*> pair will only be kept if it has more than 40% horizontal genome coverage (*fraction_covered*) and 5X vertical genome coverage (*mean_coverage*). Furthermore, only "sufficiently prevalent" species with "sufficiently many" samples (by default *sample_counts* > 2) are eligible for population SNV analyses (across samples). Therefore, different species may have different lists of relevant samples. For each genomic site, a sample is "relevant" if the corresponding site depth falls between the user-defined range, otherwise it is ignored for the pooled-SNV compute. Therefore, different genomic sites from the same species may have different panels of "relevant samples". And genomic site prevalence can be computed as the ratio of the number of relevant samples for the given site over the total number of relevant samples for the given species.

There are three main steps to compute and report population SNV in MIDAS2. First, for each site, MIDAS2 determines the set of alleles present across all relevant samples. Specifically, for each allele (A, C, G, T), *merge_snps* subcommand (1) tallies the sample counts (*sc*) of relevant samples containing corresponding allele, and (2) sums up the read counts (*rc*) of the corresponding allele across all the relevant samples. Second, population major and minor alleles for a single site can be computed based on the accumulated read counts or sample counts across all relevant samples. The population major allele refers to the most abundant/prevalent allele, and the population minor allele refers to the second most prevalent/abundant allele. Third, MIDAS2 collects and reports the sample-by-site matrix of the corresponding (1) site depth and (2) allele frequency of the population minor allele for all the relevant samples.

Chunkified Pileup Implementation

MIDAS parallelized pileup work by species, and because some species required more work than others, this resulted in a few CPU cores working longer while the rest sat idle waiting. So MIDAS2 subdivided the work for those large species into smaller units that we called chunks, by splitting the species' genomes into segments. We size the chunks so they take reasonable time (default *chunk_size* = 1000000). If chunks are too big, we will have CPU cores sitting idle while others finish running. If chunks are too small, the per-chunk setup costs will be high. The chunkified pileup algorithm can sweep through a large data set coherently, ensuring data locality so the active chunks fit into available filesystem cache, and it therefore achieves better utilization.

To compute the per-chunk population SNV, all the pileup results of corresponding sites across all the samples need to be read into memory. Therefore, at any given moment, up to total CPU cores * total number of sites per chunk * total number of the relevant samples pileup results will be read into RAM. For a highly prevalent species, MIDAS2 dynamically adjusts to a smaller chunk size (*--robust_chunk*). There is a trade-off between the chunk size, the running time and the memory usage. Users can customize the chunk size according to their computing environment. For example, constant RAM usage can be achieved despite an increasing number of samples with dynamic chunk sizes. When all chunks from the same species finish processing, chunk-level pileup results will be merged into species-level pileup results. Below is the pseudocode for the chunkified population SNV implementation.

```
def process(species_id, chunk_id):
    if chunk_id == -1:
        collect_chunks()
    chunk_worker()
def chunk_worker():
    for sample in list_of_relevant_samples:
        accumulate()
    call_population_snps()
    write_population_snps()
multiprocessing_map(process,list_of_species,
num_cores)
```

This implementation makes reference-based population SNV analysis across thousands of samples possible. We also implemented chunkified analysis for the MIDAS2 CNV module so that species with particularly large number of genes in their pangenomes can be split into multiple parallel jobs.

Consensus and Population SNVs for a Standardized Microbial Community Reveal that Reference Genome Selection and Post-alignment Filters Determine Metagenotyping Accuracy

We compared the consensus sequences reported by three metagenotyping pipelines using three metagenomes from aliquots of the same ZymoBIOMICS Microbial Community Standard product (catalog no. D6300, BioProject PRJNA648136). No genetic differences were expected between these samples. For each species, SNVs were called by comparing all pairs of metagenomes; and we computed the pairwise consensus SNVs and population SNVs to assess the false positive rates of each method as in (Olm et al. 2021).

Analysis. Three metagenotyping pipelines that use alignment to reference databases were compared: MIDAS2, inStrain, and metaSNV v2. We ran inStrain with the Zymo reference genomes as in (Olm et al. 2021), metaSNV v2 with proGenomes 2 as in (Mende et al. 2020), and MIDAS2 with MIDAS DB v1.2 for the purpose of reproducibility with results in (Olm et al. 2021). For the post-alignment filters, we used the default or recommended filter parameters by each tool (Supplementary Table S4). To evaluate the role of a well-matched reference database, we also ran MIDAS2 with the Zymo reference genomes, while keeping everything else unchanged. This allowed for a direct comparison of the post-alignment filters deployed in inStrain and MIDAS2, as well as a direct assessment of the reference database between MIDAS DB v1.2 and Zymo reference genomes. All the tools report horizontal genome coverage, upon which species presence is called. The same set of variant-calling filters as inStrain were applied to all the tools. Last, pairwise consensus-based SNVs (consensus SNVs) and population SNVs were computed. Simply put, consensus SNVs refer to two major alleles that are different between a pair of samples, whereas population SNVs refer to sites that differ (neither major or minor allele match) between a pair of samples.

Database. For the eight bacterial Zymo reference genomes, we found the corresponding species in MIDAS DB v1.2 and proGenomes2 based on the highest Average Nucleotide Identity (ANI) (Jain et al. 2018). It is worth mentioning that for Lactobacillus fermentum and Salmonella enterica, there are two pairs of highly closely related strains in the proGenomes2: 98.8% and 98.5% (Supplementary Table S6). We also reported the genome quality of all the representative genomes in all three databases using checkM (Parks et al. 2015) (Supplementary Table S7).

Results. The three aliquot samples originated from the same microbial population, therefore any observed SNVs could be a product of (1) read misalignment: some regions of the reference genome erroneously recruit reads originating from other genome regions or even other species; (2) failure of the post-alignment filters to recognize the misalignments as such; or (3) microdiversity created in the lab.

MIDAS2 reported more accurate consensus sequences compared to inStrain when both tools were run using the Zymo reference genomes matched to the strains in the metagenomes (Figure 1B). However, the accuracy of MIDAS2 was much inferior when the Zymo reference database was replaced by MIDAS DB v1.2, in which the representative genomes are less similar to the community and also suffer from fragmented assemblies. Population SVNs followed the same pattern (Supplementary Figure S6). This shows that the reference genomes are the major determinant of accuracy for consensus SNVs, explaining a previous report of poor performance for MIDAS (Olm et al. 2021). As suggested by Olm et al., a good representative genome has the following characteristics: high quality contiguous sequence and a high degree of shared gene content with the taxa it is meant to represent.

On the other hand, with its stringent post-alignment filters (e.g., recruiting only uniquely aligned reads), metaSNV v2 only reported metagenotypes for five of the eight species in the mock community. Three species did not pass its default 40% horizontal coverage threshold (Supplementary Table S8). A few reasons can explain this failure of metaSNV v2. First, there is not an appropriate representative genome of the species Bacillus subtilis in the proGenomes 2; the closest genome has average nucleotide identity (ANI) = 92.54% (*txid* 224308). Second, there are two very closely related genomes in the proGenome 2 database for the other two species: Lactobacillus fermentum (ANI=98.83%) and Salmonella enterica (ANI=98.53%), which led to extremely low numbers of uniquely alignable reads for these two species. Since metaSNV v2 requires uniquely mapped reads, these two species have low horizontal genome coverage (0.04% and 30% of the whole genome, respectively) and hence many fewer sites metagenotyped. Third, metaSNV v2 only reports non-reference variants in the output. Therefore, significantly fewer genomic sites were metagenotyped and further compared between a pair of samples by metaSNV v2. As a result, metaSNV v2 ran faster than the other two tools and reported fewer false positive SNVs (Supplementary Table S5). For the 1097 samples from the PREDICT study (PRJEB39223), the analysis-ready BAM file of MIDAS2 is 1.53 times larger than metaSNV v2, again due to the different post-alignment filters. Thus, metaSNV v2 generates few false positive SNVs and runs efficiently, but it has lower sensitivity than MIDAS2 and inStrain.

Summary. To summarize, we demonstrated building a high-quality genome database and utilizing post-alignment filters that balance false positive versus false negative SNVs determine metagenotype accuracy.

MIDAS2 Reference Database Target Layout and Construction

MIDAS Reference Database (MIDAS DB) refers to a set of custom files needed to run MIDAS2. There are three components in a MIDAS DB: rep-genome database, SCG marker database, and pan-genome database (Supplemental Figure S3). In each part, data is organized by species. For a given species, the rep-genome database contains all representative genomes of that species annotated using Prokka (Seemann 2014), and the SCG marker database is built on identified homologs of 15 universal SCGs from the representative genomes. The pan-genome database refers to the set of non-redundant genes within all genomes from that species (Commichaux et al. 2021), clustered at 99% sequence identity using vsearch (Rognes et al. 2016).

The original release of MIDAS provided a default bacterial reference database (MIDAS DB v1.2), constructed from a collection of 5952 bacterial species clusters representing 31,007 high-quality bacterial genomes. With the rapid growth of the number of sequenced microbial genomes, particularly with the addition of metagenome-assembled genomes (MAGs) from diverse habitats, it is necessary to update the MIDAS DB.

For MIDAS2, we took advantage of several published collections of prokaryotic genome collections (UHGG and GTDB) in which genomes were already clustered into species groups. We developed a new database infrastructure that is geared to run on AWS Batch and S3, achieving elastic scaling for database construction for each of these large genome collections. Specifically, MIDAS DB construction can be executed in AWS using hundreds of instances, depositing built products in S3. For example, the species pan-genome for all 47,894 species of GTDB r202 was built in roughly one week at a cost of $80K, using 100 r5d.24xlarge instances. The new database infrastructure reads in a table-of-contents (TOC) file containing genome-to-species assignments for all the genomes and a choice of representative genome for each species cluster. Six-digit numeric species ids are randomly assigned and stored in the corresponding metadata file (*metadata.tsv*). MIDAS2 users can also build a new MIDAS DB locally for a small collection of representative genomes of interest. New databases enable selection of rep-genomes that match the sample more closely than our pre-computed MIDASDBs do (Fig. 1B).

UHGG Separable/Inseparable Species

Many species have a closely related species with pairwise average nucleotide identity (ANI) near the species boundary (e.g., 95% ANI). For example, in the UHGG v1 database, there are 981 species with closest pairwise ANI higher than 92%. We explored how effective SCGs are in separating the species in the UHGG database. For a given SCG in a given species, we compute all 31-mers and compare them to 31-mers present in all species. We define the marker as separable if it has >100 unique 31-mers. We then define a species as separable if more than 50% of its 15 SCGs are separable. We found that there are 3,956 separable species and 689 inseparable species (Supplementary Tables S13 and S14). The majority (76%) of inseparable species are from the *Collinsella* genus. The representative genomes of the 3,956 separable species were used as the reference database for the PREDICT analysis in this study.

Bioinformatics Processing of Publicly Down-loaded Samples

Metagenomic sequencing reads from an inflammatory bowel disease cohort (NCBI accession: PRJNA400072) were pre-processed using Sunbeam (Clarke et al. 2019), and 211 samples with more than 5 million reads were used for benchmarking. The benchmark work was done on a m5.16xlarge or m5.24xlarge EC2 instance, depending on the CPU counts needed. Metagenomic sequencing reads from the PREDICT cohort (NCBI accession: PRJEB39223) were pre-processed using Sunbeam, and 1,097 samples with more than 5 million reads were used in our analysis. The benchmark of MIDAS2 and metaSNV v2 was done on a r5-24xlarge instance. The bioinformatics details of the ZymoBIOMICS benchmarking experiment are shown in Supplementary Table S4. The parameters used in the PREDICT analysis are shown in Supplementary Table S10.

Parameters for Paired-end Read Alignment in MIDAS2

During Bowtie2 read alignment, the default maximum fragment length is 500 base pairs (bp). With this setting, any read pairs sequenced from DNA fragments longer than 500 bp are labeled as not properly aligned

(*is_proper_pair* = FALSE), and the reported template length may also be incorrect (equal to zero) (Supplementary Figure S12). Therefore, to properly use the paired-end option of the single-sample SNV module, it is crucial to set the *-X* (maximum fragment length) to an appropriate value for the metagenomes being analyzed (default =5,000 bp), even if longer maximum fragment length will make the alignment slower.

Runtime Comparison with inStrain and metaSNV

The across-samples pooled SNVs analysis of both inStrain and metaSNV require aligning the samples to the same genome database, while MIDAS2 builds sample-specific genome databases. Furthermore, inStrain takes a raw BAM file as input, metaSNV takes a filtered BAM file as input, and MIDAS2 takes unaligned reads as input. Therefore, in order to mount a fair comparison of runtime between the three methods, we precompiled a list of 113 metagenomic samples from the IBD cohort and 21 species that are abundant (vertical genome coverage >= 5X, horizontal genome coverage >= 40%) and prevalent (present in more than 40% of samples) in in these samples. This enabled us to limit the benchmark to the same species with all three methods. Next, we ran bowtie2 to generate an unfiltered BAM file as input to inStrain plus a filtered BAM file as input to metaSNV. Finally, we ran MIDAS2 with alignment filters for comparison to inStrain and without filters for comparison with metaSNV. For each species and each method, we computed pooled or population SNV allele frequency tables, tracking the time from inputs to the tables. All tools were tested with 32 CPUs on a m5.16xlarge EC2 instance. We found that MIDAS2 is 2.3 times faster than inStrain v1.6.3 (Supplemental Table S13) and 1.19 times slower then metaSNV v2 (Supplemental Table S14). However, it is worth noting that inStrain additionally computed many microdiversity metrics, and metaSNV does not generate single-sample SNV results.

Simulations with Multiple Strains

To investigate when MIDAS2 can detect the minor allele for a sample with two strains, we conducted a series of simulations with two strains of the same species at varying abundances. We simulated reads from two NCBI strains of Phocaeicola-dorei (MIDASDB species_id 102478): Phocaeicola-dorei-5-1-36/D4 (GCF_000158335.2) and Phocaeicola-dorei-DSM-17855 (GCF.013009555.1). We determined the correct genotypes for these strain mixture by using whole-genome alignments via nucmer (*true_sites*) (Marcais et al. 2018). Given the high within-species ANI between the two mixture strains, there are 16603 ALT sites and 4193849 REF sites, where ALT refers to genomic sites where the two strains have different alleles, and REF refers to sites where the strains have the same allele. MIDAS2 single-sample SNV analysis was run on each simulated metagenome. Only sites covered by more than five reads were genotyped. To evaluate performance, we computed the precision and recall of all genotyped sites compared to the true genotypes. We observed that REF sites can be genotyped with high precision and recall whenever the less abundant strain has at least 1x horizontal genome coverage, while 5x coverage is needed for ALT sites (Supplementary Table S13).

Quasiphasable Species Model

We applied the model from Garud, Good et al. (Garud et al. 2019) to the PREDICT study to determine whether each species in each metagenomic sample was quasi-phasable (QP) or not (i.e., one dominant strain versus colonization by multiple bacterial lineages). The model uses synonymous sites in genes of the core genome of a given species. If the fraction of these sites with intermediate allele frequencies is high, this is taken as evidence for a strain mixture. We observed that the vast majority of these sites and all sites genome wide are biallelic or fixed. For example, on average 0.00609% of sites have more than two alleles for PREDICT samples with one dominant strain of *Bacteroides_B dorei*, and 0.0342% sites have more than two alleles for PREDCIT samples with multiple strains of *Bacteroides_B dorei*.

Bioinformatics. We use similar sample and site filters for population SNVs as in (Garud et al. 2019). Specifically, we filter the per species *snps_info.tsv* and *snps_freqs.tsv* files as follows:

- Minimal per sample median site depth of bi-allelic SNVs from protein coding sequences ($\bar{D}$) is 20.
- Only include 4-fold degenerate synonymous sites
- Sample site depths must be between .3 * $\bar{D}$ and 3 *$\bar{D}$
- Minimal site depth is 10
- Minimal site prevalence is 5

This produces a filtered SNVs allele frequency file.

QP calculation. For each species, we estimated whether each sample is QP as follows:

(1) For each non-intermediate site, we defined population major allele direction ($\bar{f}_{l_{dir}}$) as 1 if the majority of the allele frequencies are higher than 0.8 and 0 if not:

$$\bar{f}_{l_{dir}} = 1 \text{ if } (\sum_{n}^{N} f_n \geq 0.8 \geq \sum_{n}^{N} f_n \leq 0.2) \text{ else } \bar{f}_{l_{dir}} = 0$$

where n is the sample index, N is the number of relevant samples for the given site.

(2) For each sample n, we computed the dominant haplotype of each non-intermediate site ($f_{n,l_{dir}}$) as 1 if the corresponding allele frequency is higher than 0.8 and 0 if not:

$$f_{n,l_{dir}} = 1 \text{ if } f_{n,l} \geq 0.8 \text{ else } f_{n,l_{dir}} = 0$$

where l is the site index.

(3) For each non-intermediate site, the population major allele frequency ($\bar{f}_l$) is computed as:

$$\bar{f}_l = \frac{\sum_n^N f_{n,l_{dir}} == \bar{f}_{l_{dir}}}{N}$$

(4) For each sample, we estimated N_D as the average genetic distance between sample n and the alleles present in the remainder of the samples ($\bar{f}_{l_{dir}}$) as:

$$N_D = \sum_{l}^{L} \bar{f}_l \text{ if } f_{n,l_{dir}} == \bar{f}_{l_{dir}} \text{ else } 1 - \bar{f}_l$$

where, l is site index, L is total number of non-intermediate sites; n is the sample index.

(5) For each sample, we computed the number of intermediate alleles over all sites ($N_<$):

$$N_< = \sum_{l}^{L} 1 \text{ if } 0.2 < f_{n,l} < 0.8 \text{ else } N_< = 0$$

(6) For each sample, if $\frac{N_<}{N_D} < 0.1$, then we say the sample is QP, because there is evidence of one dominant strain with sufficiently high coverage and sufficiently low rates of intermediate alleles.

PCoA and Manhattan distance

Manhattan distance was calculated based on the filtered SNV site-by-sample allele frequency matrix to evaluate the dissimilarity between samples. Principal Coordinate Analysis (PcoA) was calculated based on the Manhattan distance matrix using the ape package (Paradis et al. 2004).

References

Almeida, A. *et al.* (2021). A unified catalog of 204,938 reference genomes from the human gut microbiome. *Nature Biotechnology*, *39*(1), 105–114.

Andreas Heger, K. J. (n.d.). *pysam*. Retrieved June 13, 2022, from https://github.com/pysam-developers/pysam

Beghini, F., McIver. *et al.* (2021). Integrating taxonomic, functional, and strain-level profiling of diverse microbial communities with bioBakery 3. *eLife*, *10*.

Bush, S. J. *et al.* (2020). Genomic diversity affects the accuracy of bacterial single-nucleotide polymorphism-calling pipelines. *GigaScience*, *9*(2).

Clarke, E. L. *et al.* (2019). Sunbeam: an extensible pipeline for analyzing metagenomic sequencing experiments. *Microbiome*, *7*(1), 46.

Commichaux, S. *et al.* (2021). A critical assessment of gene catalogs for metagenomic analysis. *Bioinformatics*.

Daillère, R. *et al.* (2016). Enterococcus hirae and Barnesiella intestinihominis Facilitate Cyclophosphamide-Induced Therapeutic Immunomodulatory Effects. *Immunity*, *45*(4), 931–943.

Danecek, P. *et al.* (2021). Twelve years of SAMtools and BCFtools. *GigaScience*, *10*(2).

Garud, N. R. *et al.* (2019). Evolutionary dynamics of bacteria in the gut microbiome within and across hosts. *PLoS Biology*, *17*(1), e3000102.

Jain, C. *et al.* (2018). High throughput ANI analysis of 90K prokaryotic genomes reveals clear species boundaries. *Nature Communications*, *9*(1), 5114.

Jiang, H. *et al.* (2015). Altered fecal microbiota composition in patients with major depressive disorder. *Brain, Behavior, and Immunity*, *48*, 186–194.

Marcais, G. *et al* (2018). MUMmer4: A fast and versatile genome alignment system. *PLOS Computational Biology*, *14*(1), e1005944.

Mende, D. R. *et al.* (2020). proGenomes2: an improved database for accurate and consistent habitat, taxonomic and functional annotations of prokaryotic genomes. *Nucleic Acids Research*, *48*(D1), D621–D625.

Nayfach, S., & Pollard, K. S. (2015). Population genetic analyses of metagenomes reveal extensive strain-level variation in prevalent human-associated bacteria. In *bioRxiv* (p. 031757).

Nayfach, S., & Pollard, K. S. (2016). Toward Accurate and Quantitative Comparative Metagenomics. *Cell*, *166*(5), 1103–1116.

Nayfach, S. *et al.* (2016). An integrated metagenomics pipeline for strain profiling reveals novel patterns of bacterial transmission and biogeography. *Genome Research*, *26*(11), 1612–1625.

Olm, M. R. *et al.* (2021). inStrain profiles population microdiversity from metagenomic data and sensitively detects shared microbial strains. *Nature Biotechnology*, *39*(6), 727–736.

Paradis, E. *et al.* (2004). APE: Analyses of Phylogenetics and Evolution in R language. *Bioinformatics*, *20*(2), 289–290.

Parks, D. H. *et al.* (2015). CheckM: assessing the quality of microbial genomes recovered from isolates, single cells, and metagenomes. *Genome Research*, *25*(7), 1043–1055.

Parks, D. H. *et al.* (2022). GTDB: an ongoing census of bacterial and archaeal diversity through a phylogenetically consistent, rank normalized and complete genome-based taxonomy. *Nucleic Acids Research*, *50*(D1), D785–D794.

Robinson, J. T. *et al.* (2011). Integrative genomics viewer. *Nature Biotechnology*, *29*(1), 24–26.

Rognes, T. *et al.* (2016). VSEARCH: a versatile open source tool for metagenomics. *PeerJ*, *4*, e2584.

Seemann, T. (2014). Prokka: rapid prokaryotic genome annotation. *Bioinformatics*, *30*(14), 2068–2069.

Truong, D. T. *et al.* (2017). Microbial strain-level population structure and genetic diversity from metagenomes.

Van Rossum, T. *et al.* (2021). metaSNV v2: detection of SNVs and subspecies in prokaryotic metagenomes. *Bioinformatics*.

Supplementary Figures

Fig S1: MIDAS2 is the only reads-to-table whole-genome based metagenotyping pipeline.

Fig S2: MIDAS2 performs strain-level metagenomics analysis through two modules.

Fig S3: MIDAS2 Reference Database (MIDAS DB) target layout and major construction steps.

Fig S4: MIDAS2 achieved better CPU utilization compared to MIDAS.

Fig S5: Database building and alignment on average take 75% of run time in the MIDAS2 single-sample SNV module.

Fig S6: ZymoBIOMICS benchmark shows reference genome selection and post-alignment filters determine metagenotyping accuracy.

Fig S7: Compute performance comparison between MIDAS and MIDAS2 for single-sample CNV module.

Fig S8: PCoA plot of two species with genetically distinct lineages across samples, yet a single dominant strain within most samples.

Fig S9: Many samples are colonized with more than one strain of *Bacteroides_B dorei*.

Fig S10: Many samples are colonized with more than one strain of *Faecalibacterium prausnitzii_G*.

Fig S11: Importance of setting proper -X for paired-ends reads alignment

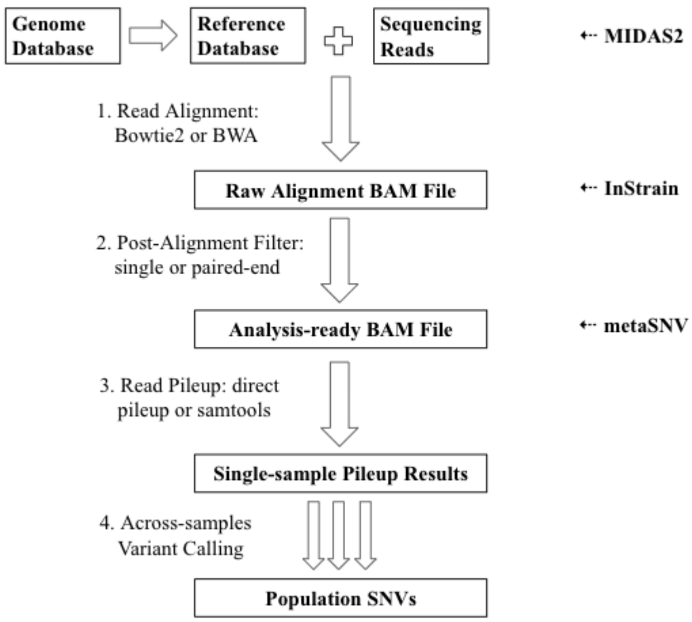

Fig S1. **MIDAS2 is the only reads-to-table whole-genome based metagenotyping pipeline**. Reference-based metagenotyping pipelines share an underlying structure, yet they differ from one another in whether or not they include code for generating and customizing the genome database to which metagenomic reads are aligned, the choice of reference genomes in the database, the reads alignment algorithm, pileup tools, post-alignment filters, as well as the input and output file contents. MIDAS2 starts from metagenomic sequencing reads and then proceeds through alignment, post-alignment filtering, pileup, and variant calling. It includes pre-computed reference databases, code for building a database, and code for customizing the database to the species present in the metagenome. The other tools leave database and alignment to the user. InStrain (Olm et al. 2021) starts from a BAM file and performs post-alignment filtering as a first step, whereas metaSNV v2 (Van Rossum et al. 2021) starts from a BAM file that has already been filtered. By integrating and automating all steps of the metagenotyping process, MIDAS2 helps to promote reproducible research.

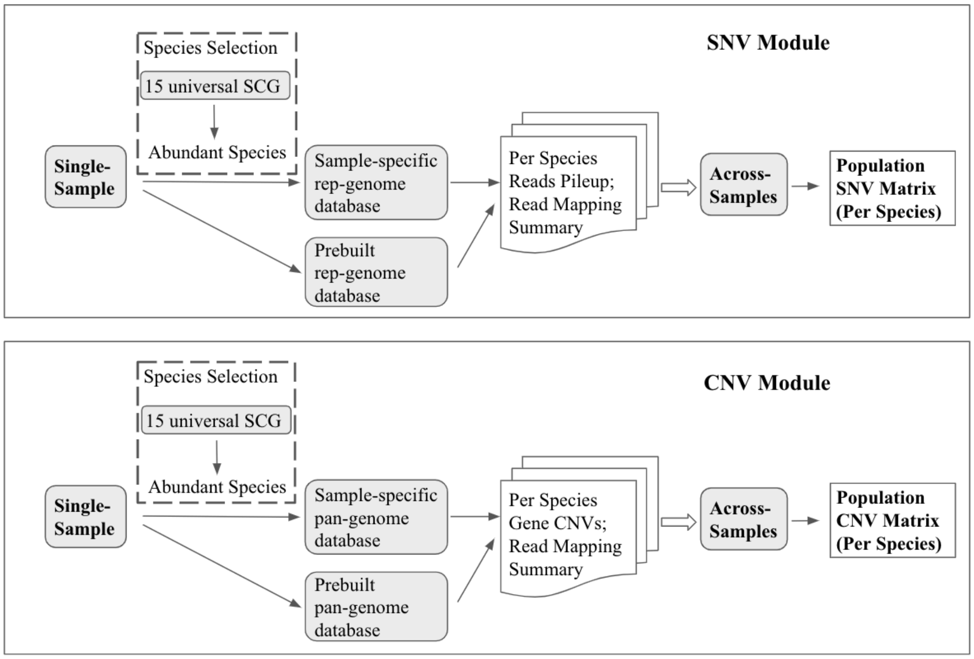

Fig S2. **MIDAS2 performs strain-level metagenomics analysis through two modules:** population single nucleotide variants analysis (SNV module) and pan-genome gene copy number variants analysis (CNV module). Each module includes two sequential steps: single-sample analysis and across-samples analysis. The single-sample step for both modules uses an estimate of which species are present in the sample to customize the sample-specific bowtie2 database. Across-sample analysis reports both the read alignment and genotyping summary, and multiple per-species sample-by-site SNV or sample-by-gene CNV matrices.

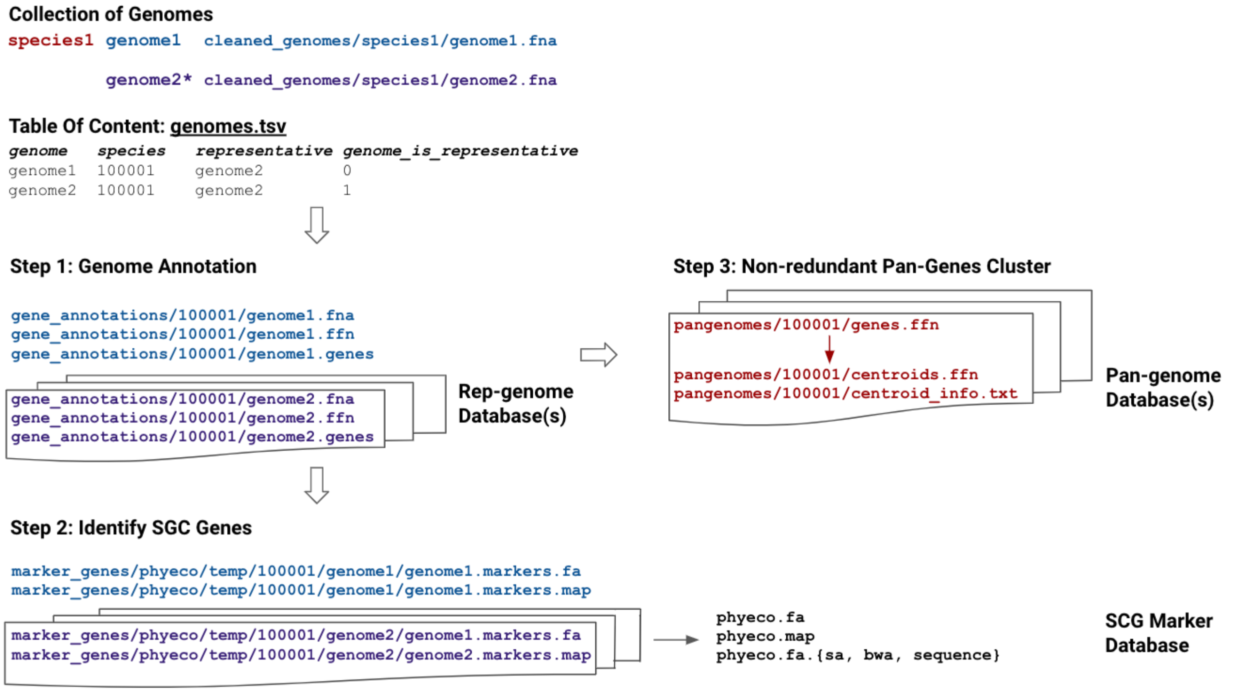

Fig S3. **MIDAS2 Reference Database (MIDAS DB) target layout and major construction steps.** There are three components in a MIDAS DB: rep-genome database, Single Copy Gene (SCG) marker database, and pan-genome database.

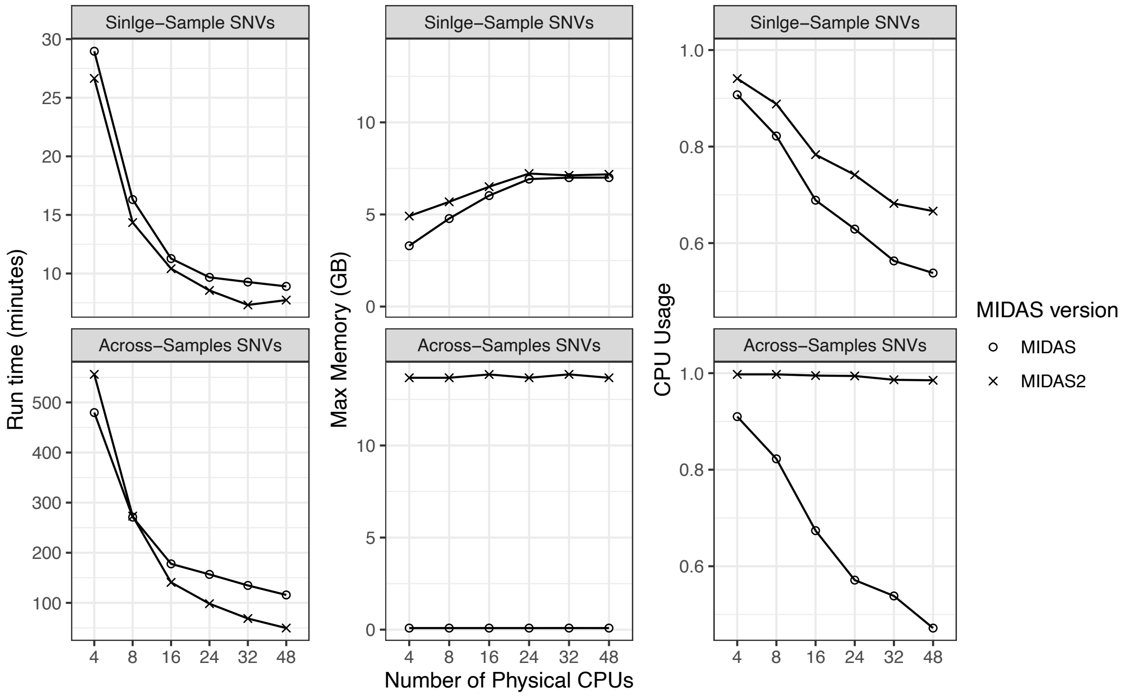

Fig S4. MIDAS2 achieves better CPU utilization compared to MIDAS. The single-sample SNV module of MIDAS2 is on average up to 1.27 times faster than MIDAS (number of CPUs = 32). This improvement is limited because 75% of the single-sample SNV analysis is spent on building sample-specific genome database, read alignment and post-alignment filtering (Supplementary Figure S6), which are outside the chunk-parallelization we implemented. The across-samples SNV module benefits more from chunk-parallelization. It is up to 2.33 times faster than MIDAS (number of CPUs = 48). CPU usage is defined as CPU percentage / number of cores. The chunk-parallelization leads to the across-samples module scaling almost linearly with the number of physical cores (tested up to 48 cores).

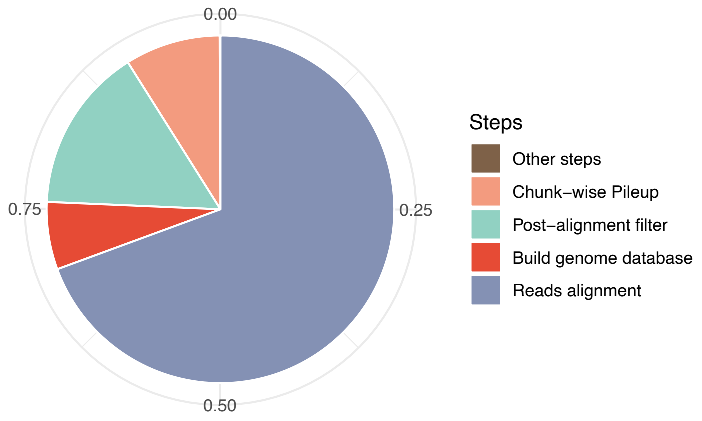

Fig S5. **Database building and alignment on average take 75 % of run time in the MIDAS2 single-sample SNV module.** We analyzed the distribution single-sample SNV module run times for 211 metagenomic samples (PRJNA400072) with 8 physical CPUs and using paired-end post-alignment filtering. Single-sample pileup is a multi-step task: build sample-specific genome database, perform read alignment, apply post-alignment filter, and do pileup.

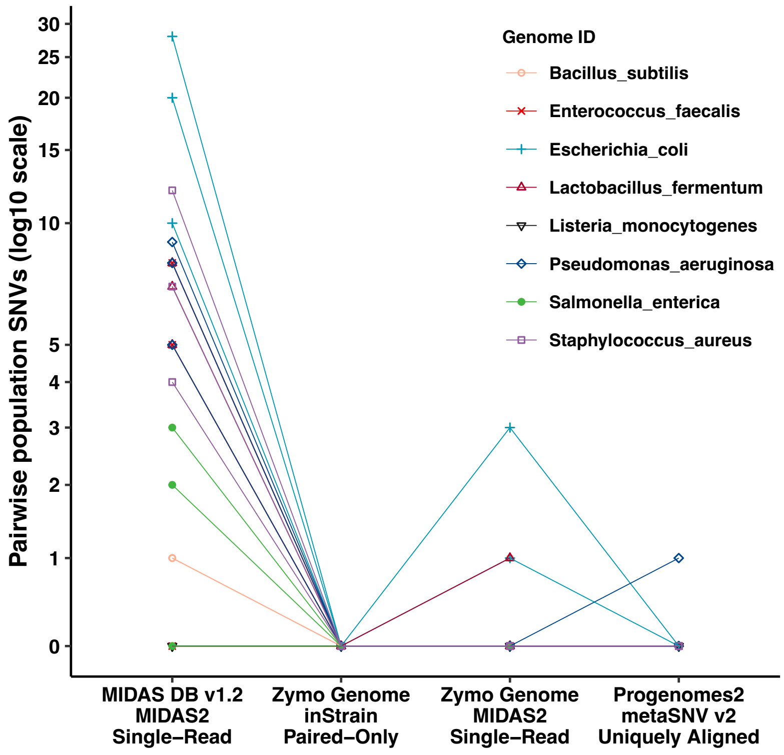

Fig S6. **ZymoBIOMICS benchmark shows reference genome selection and post-alignment filters determine metagenotype accuracy**. MIDAS2 with Zymo reference genome reported more accurate consensus SNVs compared to inStrain, yet the accuracy of MIDAS2 was inferior when the Zymo genomes were replaced by a more diverse database MIDAS DB v1.2 (genomes for many more species, genomes of the eight on-target species diverged from those in the metagenome). Fewer genomic sites were metagenotyped by metaSNV v2 due to its strict alignment uniqueness filters, resulting in the lowest false positive rates. However, only five out of the eight species were metagenotyped by metaSNV v2, compared to all eight for inStrain and MIDAS2 (Supplemental Table S9). Across methods, population SNVs were more accurate than consensus SNVs. These results show that metagenotype precision and recall depends upon building a good reference genome database and utilizing appropriate post-alignment filters. More details see Supplemental Notes.

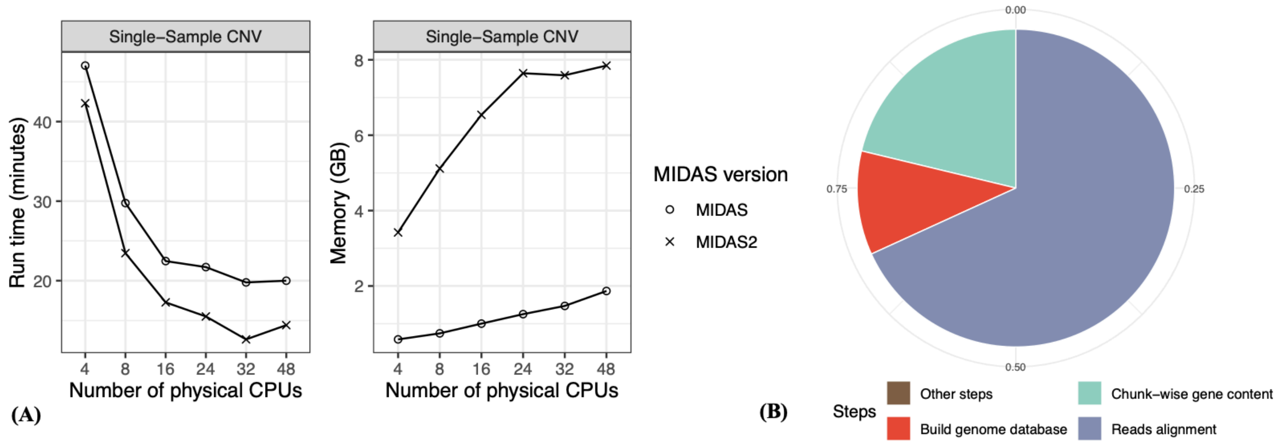

Fig S7. Compute performance comparison between MIDAS and MIDAS2 for single-sample CNV module. This benchmark was performed using 211 samples (PRJNA400072). **A:** The single-sample CNV module of MIDAS2 is on average up to 1.57 times faster than that of MIDAS (number of cores = 32). **B:** This minor improvement is because 80% of the single-sample CNV module is spent on building a sample-specific genome database plus read alignment.

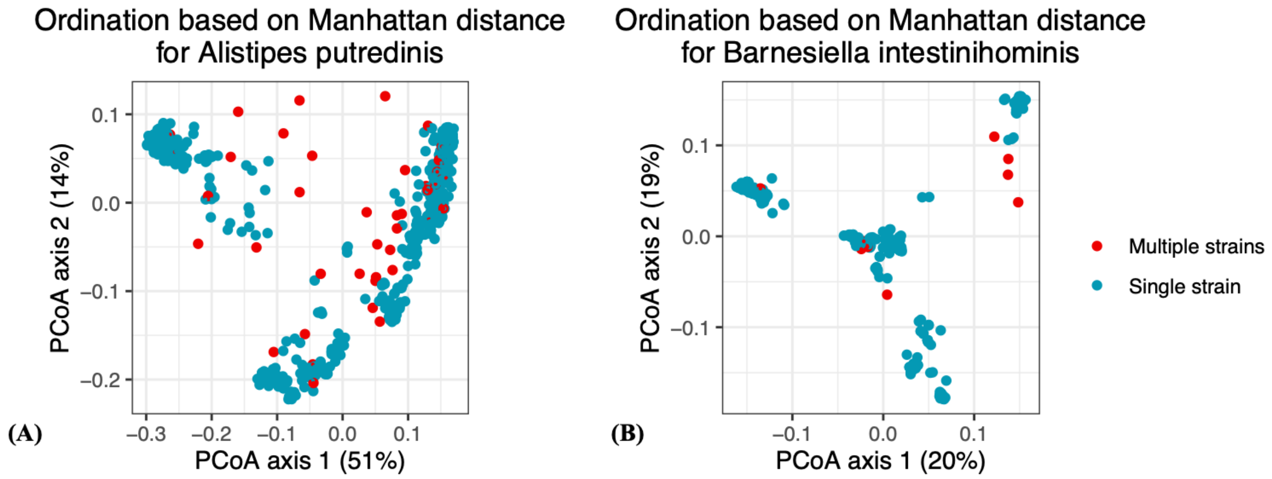

Fig S8. PCoA plot of two species with genetically distinct lineages across samples, yet a single dominant strain within most samples. PCoA was performed based on the pairwise Manhattan distance between samples computed from the population SNV minor allele frequency matrix of one single species. Each dot in the PCoA plot represents one sample, and the distance between a pair of dots represents the genetic similarity of that species in the two metagenomes. A: *Alistipes putredinis*, a species which is overly abundant in depression (Jiang, H. et al. 2015) patients. There appear to be two or three distinct clusters of samples, each comprised primarily of samples with a single strain. B: *Barnesiella intestinihominis* (Daillère, R. et al. 2016), a species associated with anti-cancer effects. There are four distinct clusters of samples.

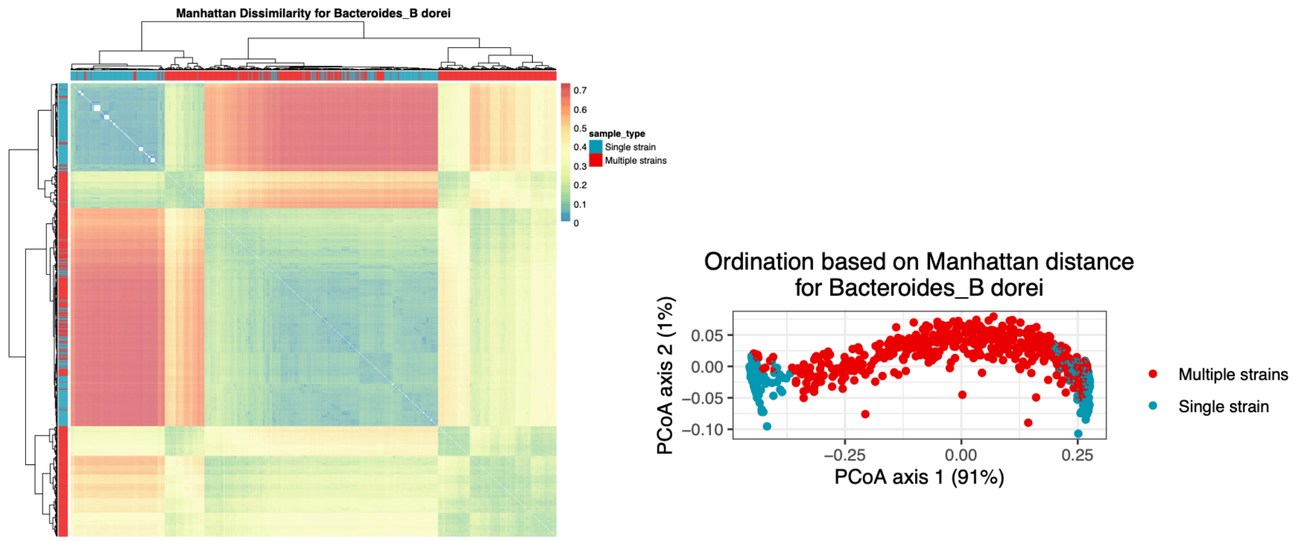

(A) (B)

Fig S9. Many samples contain more than one strain of *Bacteroides_B dorei*. 62% of the 932 PREDICT samples with *B. dorei* have allele frequency spectra consistent with two or more distinct lineages colonizing the host. Samples with one dominant strain of *B. dorei* form two genetically distinct clusters, and the rest of the samples are intermediate between these, consistent with colonization by strains from both clusters. **A:** Heatmap of pairwise Manhattan distances between PREDICT samples. **B:** PCoA plot based on the Manhattan distances.

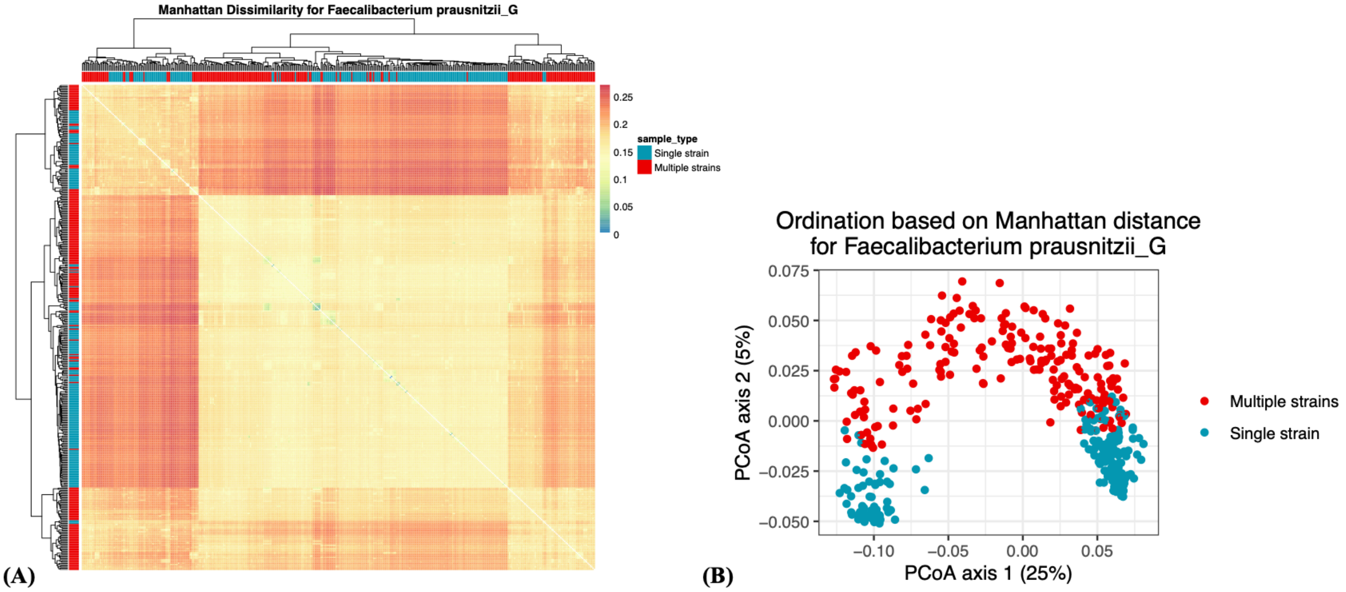

(A) (B)

Fig S10. Many samples contain more than one strain of *Faecalibacterium prausnitzii_G*. 49% of the 401 PREDICT samples with *F. prausnitzii_G* have allele frequency spectra consistent with two or more distinct lineages colonizing the host. Samples with one dominant strain of *F. prauznitzii_G* form two genetically distinct clusters, and the rest of the samples are intermediate between these, consistent with colonization by strains from both clusters. **A:** Heatmap of pairwise Manhattan distances between PREDICT samples. **B:** PCoA plot based on the Manhattan distances.

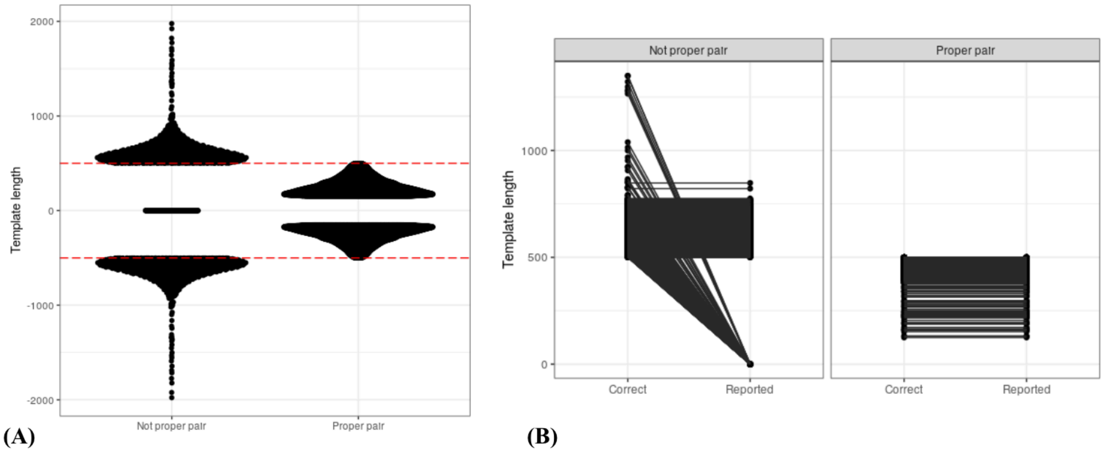

(A) (B)

Fig S11. **Importance of setting proper *-X* for paired-end read alignment**. Simulated paired-end reads with average fragment length 600 bps were aligned to the representative genome with default maximum fragment length of Bowtie2 (500 bps). **A:** We observed that read pairs sequenced from DNA fragments longer than 500 bps are often labeled as "Not proper pair" (*is_proper_pair* = FALSE). **B:** We also observed that the template length reported by Bowtie2 for read pairs originating from longer fragments could be falsely reported as 0. Therefore, it is crucial to set a long enough *-X* to ensure reads from longer fragments will be properly aligned, even though it will make alignment slower.

Supplementary Tables

Table S1: MIDAS2 software updates

Table S2: Comparison of prokaryote species pan-genome databases.

Table S3: Comparison of gene profiling tools for metagenomes.

Table S4: ZymoBIOMICS benchmarking experiment.

Table S5: Consensus SNVs and population SNVs results for the ZymoBIOMICS benchmark.

Table S6: Corresponding species in MIDAS DB v1.2 and proGenomes 2 for 8 bacterial ZymoBIOMICS genomes.

Table S7: Genome quality of Zymo genomes and corresponding representative genomes in MIDAS DB v1.2 and proGenomes 2.

Table S8: Comparison of average horizontal genome coverage in a single sample in Zymo benchmark experiment.

Table S9: Comparison of average fraction of bases compared in a pair of samples in the Zymo benchmark experiment.

Table S10: Bioinformatics Details of experimental design for PREDICT analysis.

Table S11: Comparison of compute resources for PREDICT benchmarking using a r5-24xlarge instance.

Table S12: Discrepancy of variant calling results with samtools and MIDAS2 at one genomic site.

Table S13: Runtime comparison of MIDAS2 with inStrain v1.6.3 for 21 species from the IBD study.

Table S14: Runtime comparison of MIDAS2 with metaSNV v2 for 21 species from the IBD study.

Table S15: Precision and recall of MIDAS2 genotyping results for simulated samples with two strains of Bacteroides_B dorei.

Table S16: 3956 separable UHGG species list.

Table S17: 689 inseparable UHGG species list.

Table S1. MIDAS2 Software Updates.

		MIDAS	MIDAS2
**MIDAS Reference Database**		1. Single MIDAS DB available.<br>2. The whole database needs to be downloaded before any analysis.	1. Multiple MIDAS DBs available.<br>2. Databases can be downloaded for specific species only.<br>3. Script for building custom databases locally or in the cloud using AWS Batch.
**Species Selection**	**Single Sample**	1. Select sufficiently abundant species by mean coverage across 15 universal, single-copy genes (SCGs).	1. Select sufficiently abundant species by median SCG coverage and the fraction of SCGs with uniquely aligned reads.
**SNV Module**	**Single Sample**	1. Parallelization across species.<br>2. Full matrix output.	1. Parallelization within and across species<br>2. Sparse matrix output.<br>3. Major new features: paired-end reads based post-alignment filter, accept existing Bowtie2 database.
	**Across Samples**	1. Parallelization across species.	1. Parallelization within and across species.<br>2. Robust chunking for the balance of memory and I/O.
**CNV Module**	**Single Sample**	1. Parallelization across species.<br>2. Linear search for gene annotation.	1. Parallelization within and across species.<br>2. Binary search for gene boundary and annotation.
	**Across Samples**	1. Single process.	1. Species parallel.

Table S2. Comparison of prokaryote species pan-genome databases.

	MIDAS DB	ChocoPhlAn 3.0	proGenomes 2
**Database Content**	Species rep-genomes and pan-genomes.<br>Full assignment of individual genes to clusters.	Species pan-proteomes, and clade-specific marker genes.	Species rep-genomes and pan-genomes.
**Used By**	MIDAS2	bioBakery 3 (Beghini et al. 2021)	metaSNV v2
**Genome Sources and Sizes**	UHGG v1: 4,644 species, 286,997 genomes (release July 2020);<br>GTDB r202:47,893 species, 258,405 genomes (release April 2021)	UniProt genomes and gene annotations: 16.8k species, 99.2k genomes (release January 2019)	NCBI Nucleotide database: 84,096 bacterial and archaeal genomes from over 12000 species (release May 2017)
**Pangenome Size**	MIDAS DB UHGG: 4,644 species pan-genomes;<br>MIDAS DB GTDB: 47,893 species pan-genomes.	10.7k HUMAnN3 pangenomes and 2,298;<br>PanPhlAn3 pagenomes*.	63 million pan-genes
**Highest Non-redundant Resolution**	99%	UniRef90	95%
**Strength**	Code to build a new database.	Clade-specific makers.	Habitat annotations.
**Weaknesses**	No functional annotation.	Unable to build custom genome databases.	Unable to build custom genome databases. No MAGs included.

* PanPhlAn3 pangenome refers to up to 200 genome sequences.

Table S3. Comparison of gene profiling tools for metagenomes.

	**MIDAS2 CNV Module**	**HUMAnN3** (Beghini et al. 2021)	**PanPhlAn3** (Beghini et al. 2021)
**Output**	Estimated per-species copy number per cell matrix.	Estimated per-species and community-level gene family abundance matrix.	Binary matrix of pangenome presence-absence.<br>Only one species at a time.
**Pan-genome Content**	Per-species centroids clustered at 99%.	Per-species centroids clustered at 90%.	Up to 200 genome sequences per species.
**Species Selection**	Median SCGs coverage > 2X	MetaPhlan3	Prior knowledge of species of interest.
**Strength**	Results are comparable across species.	Reconstruct metabolic pathways in the community.	A complementary form of strain analysis.

Table S4. ZymoBIOMICS benchmarking experiment.

**Pipeline**	**Genome Database**	**Database Customization**	**Aligner**	**Post-Alignment Fiter**	**Species Presence Criteria**	**Variant Calling Filter**
**MIDAS2**	MIDAS DB v1.2	Median SCG Coverage > 1X	Bowtie2	- MAPQ >= 20<br>- MAPID >= 0.94	Horizontal Genome Coverage >= 40%	- *site_depth* >= 5<br>- *allele_frequency* >= 0.05<br>- *major_allele_read_counts* >= 5<br>- *minor_allele_read_counts* >= coverage dependent null model (Olm et al. 2021)<br>- Discard ambiguous sites with tied reads counts
**MIDAS2**	Zymo Reference	N/A				
**inStrain**				- MAPQ > -1<br>- MAPID > 0.95		
**metaSNV v2**	proGenomes 2		BWA	- Mapping length >= 45 bp<br>- MAPID >= 0.97<br>- Uniquely mapped reads only*		

* We followed the post-alignment filter suggested by metaSNV v2 manual (https://github.com/metasnv-tool/metaSNV/blob/master/documentation/metaSNV_v2_manual.pdf)

Table S6. Corresponding species in MIDAS DB v1.2 and Progenomes 2 for 8 bacterial Zymo genomes.

**Zymo Genome (catalog no. D6300)**	**MIDAS Genome**	**Progenomes2 Genome**	**ANI between Zymo and Progenomes2 genomes**	**ANI between Zymo and MIDAS DB genomes**
Bacillus_subtilis	*Bacillus_subtilis_57806*	224308	92.54	100
Enterococcus_faecalis	*Enterococcus_faecalis_56297*	226185	98.66	98.78
Escherichia_coli	*Escherichia_coli_58110*	155864	97.6	98.87
Lactobacillus_fermentum	*Lactobacillus_fermentum_54035*	334390	98.89	99.25
Lactobacillus_fermentum	*Lactobacillus_fermentum_54035*	712938	98.8306	99.25
Listeria_monocytogenes	*Listeria_monocytogenes_53478*	1027396	99.51	99.71
Pseudomonas_aeruginosa	*Pseudomonas_aeruginosa_57148*	287	99.13	99.05
Salmonella_enterica	*Salmonella_enterica_58156*	90371	98.6	98.57
Salmonella_enterica	*Salmonella_enterica_58156*	1412471	98.5308	98.57
Staphylococcus_aureus	*Staphylococcus_aureus_56630*	1280	98.51	99.08

Table S8. Comparison of average horizontal genome coverage in a single sample in Zymo benchmark experiment.

Metagenotyping Pipeline		MIDAS2*	InStrain	MIDAS2	metaSNV v2
Reference Genome Database		Zymo Reference	Zymo Reference	MIDAS DB v1.2	proGenomes2
Zymo Reference Genome	*Bacillus_subtilis*	0.988	0.998	0.998	0.071
	Enterococcus_faecalis	0.981	1.000	0.762	0.784
	Escherichia_coli	0.991	1.000	0.927	0.648
	Lactobacillus_fermentum	0.980	1.000	0.906	0.039
	Listeria_monocytogenes	0.979	1.000	0.970	0.888
	Pseudomonas_aeruginosa	0.992	1.000	0.916	0.896
	Salmonella_enterica	0.990	1.000	0.905	0.295
	Staphylococcus_aureus	0.991	1.000	0.913	0.410

* MIDAS2 only considers sites covered with at least two reads when compute the horizontal genome coverage.

Table S9. Comparison of average fraction of bases compared in a pair of samples in the Zymo benchmark experiment.

Metagenotyping Pipeline		MIDAS2	inStrain	MIDAS2	metaSNV v2
Reference Genome Database		Zymo Reference	Zymo Reference	MIDAS DB v1.2	proGenomes2
Zymo Reference Genome	*Bacillus_subtilis*	0.987	0.998	0.998	N/A
	Enterococcus_faecalis	0.98	1	0.761	0.006
	Escherichia_coli	0.968	0.977	0.924	0.008
	Lactobacillus_fermentum	0.974	1	0.9	N/A
	Listeria_monocytogenes	0.978	1	0.969	0.002
	Pseudomonas_aeruginosa	0.992	1	0.916	0.004
	Salmonella_enterica	0.978	0.99	0.903	N/A
	Staphylococcus_aureus	0.986	0.996	0.91	0.003

Table S10. Bioinformatics Details of experimental design for PREDICT analysis.

	**MIDAS2**	**metaSNV v2**
**Reference Database**	3956 UHGG species (See Supplemental Note)	
**Database Customization**	- median SCG coverage > 2X<br>- uniquely mapped fraction > 0.5	N/A
**Read Mapping**	Bowtie2<br>- Global alignment<br>- Fragment length: 1000 (See Supplementary Figure S12)	BWA<br>- Local alignment
**Post-alignment Filter**	- MAPQ >= 2<br>- Paired-end based post-alignment filter<br>- MAPID >= 0.94	- Mapping length >= 45 bp<br>- MAPID >= 0.97<br>- Uniquely mapped reads only
**Input**	One analysis-ready BAM file per sample	
**Variant Calling Filters**	- Default MIDAS2 parameters	- Default metaSNV parameters
**Species and Sample Selection**	- Horizontal genome coverage >= 40%<br>- Vertical genome coverage >= 5X<br>- Species prevalence >= 50%	
**Number of cores**	48 (r5-24xlarge instance)	
**Output**	44 species were metagenotyped for population SNVs. All SNVs are reported.	14 species were metagenotyped for population SNVs. Only non-reference SNVs are reported.

Table S11. Comparison of compute resources for PREDICT benchmarking using a r5-24xlarge instance.

	**MIDAS2**	**metaSNV v2**
**Analysis-ready BAM File Size**	1.80 G per sample	1.18 G per sample
**Elapsed Time**	**Single-sample run 00:03:58 per sample**	SNV calling: 18:15:29
	**Across-samples merge: 16.2 M per species**	SNV filtering: 1:36:23
**Max Memory**	Single sample run: 4.17 G	SNV calling: 4.09 G
	Across samples merge: 17G	SNV filtering: 0.015 G

Table S12. Discrepancy of variant calling results with samtools and MIDAS2 at one genomic site.

	**Command**	**Site Depth**	**Major allele**	**Major allele frequency**	**Allele type**
**No Filter**	IGVtools direct visualization	803	G	0.91	N/A
**Same set of filters (*-q 20 -Q 30*)**	*bcftools mpileup* and *bcftools call*	247	G	1.0	Fixed
	MIDAS2 direct reads pileup	723	G	0.91	Bi-allelic

Table S13. Runtime comparison of MIDAS2 with inStrain v1.6.3 for 21 species from the IBD study (with post-alignment filters).

	MIDAS2 v1.0.2	inStrain v1.6.3
Single-Sample	11.64 hours	15.07 hours
Across Samples	22.47 minutes	12.48 hours
Total Runtime	12.01 hours	27.55 hours

Table S14. Runtime comparison of MIDAS2 with metaSNV for 21 species from the IBD study (without post-alignment filters).

	MIDAS2 v1.0.2	metaSNV v2.0.4
Single-Sample	41.20 minutes	No results reported.
Across Samples	22.47 minutes	53.50 minutes
Total Runtime	63.67 minutes	53.50 minutes

Table S15. Precision and recall of MIDAS2 genotyping results for simulated samples with two strains of *Bacteroides_B dorei*. Precision was computed as the number of correctly genotyped sites / total number of genotype-able sites. Recall was computed as the number of correctly genotyped sites / total number of *true_sites*.

Phocaeicola-dorei -5-1-36/D4	Phocaeicola-dorei -DSM-17855	Strain Ratio	Type	Precision	Recall
20X	1X	0.05	ALT	0.003	0.003
	1X	0.05	REF	1	0.987
	5X	0.25	ALT	0.779	0.758
	5X	0.25	REF	1	0.992
	10X	0.5	ALT	0.839	0.833
	10X	0.5	REF	1	0.993
	15X	0.75	ALT	0.855	0.851
	15X	0.75	REF	1	0.994
	20X	1	ALT	0.863	0.859
	20X	1	REF	1	0.994
